# Atypical Overlap Presentation in Guillain-Barré Syndrome: Lessons From a Case Report

**DOI:** 10.7759/cureus.83517

**Published:** 2025-05-05

**Authors:** Arpita Sharma, Saurabh Singhal, Vishal Vishnoi, Yashendra Sethi

**Affiliations:** 1 Department of Internal Medicine, Subharti Medical College, Meerut, IND

**Keywords:** bickerstaff encephalitis, guillain-barré syndrome, ivig, miller fisher syndrome, overlap syndrome

## Abstract

Guillain-Barré syndrome (GBS) is an acute, immune-mediated polyradiculoneuropathy characterized by rapidly progressive limb weakness and areflexia. While the classical form is well-recognized, variants such as Miller Fisher syndrome (MFS) and Bickerstaff brainstem encephalitis (BBE) also exist, each with distinct clinical features. Rarely, these syndromes may present in combination, forming GBS-MFS-BBE overlap syndromes. These atypical presentations pose significant diagnostic challenges that may delay the initiation of appropriate treatment.

We report the case of a 60-year-old male who initially presented with pain and weakness of the left upper limb, which progressed rapidly over 72 hours to symmetrical quadriplegia, bulbar dysfunction (including dysphagia and dysarthria), complete ophthalmoplegia, and altered sensorium suggestive of encephalopathy. Neurological examination revealed areflexia and bilateral facial weakness. Initial nerve conduction studies (NCSs) indicated a possible axonal plexopathy; however, follow-up NCSs performed on day 5 showed findings consistent with acute inflammatory demyelinating polyneuropathy. Given the combination of ophthalmoplegia, ataxia, encephalopathy, and demyelinating features, the diagnosis of a GBS-MFS-BBE overlap syndrome was made.

The patient was treated with intravenous immunoglobulin (IVIG) at a total dose of 2 g/kg administered over five days. Due to progressive respiratory failure, he required mechanical ventilation. Supportive care included intensive monitoring and physiotherapy. Neurological recovery was gradual, with successful weaning from the ventilator by day 25. At discharge on day 30, the patient had improved to a Medical Research Council (MRC) grade of 3/5 in all four limbs.

This case illustrates the clinical complexity and diagnostic uncertainty associated with GBS-MFS-BBE overlap syndromes. Early recognition and prompt initiation of immunotherapy, such as IVIG, are essential to improving outcomes and reducing long-term morbidity. Clinicians should maintain a high index of suspicion for atypical features, particularly in rapidly evolving neuromuscular presentations.

## Introduction

Guillain-Barré syndrome (GBS) represents the most common cause of acute flaccid paralysis worldwide, with an annual incidence of 0.8-1.9 cases per 100,000 population [1]. The classic form, acute inflammatory demyelinating polyradiculoneuropathy (AIDP), accounts for approximately 90% of cases in Western countries, while axonal variants predominate in Asia. The annual incidence of GBS in India is reported to be around 1-2 per 100,000 people [2]. The syndrome typically manifests one to three weeks following a respiratory or gastrointestinal infection, with Campylobacter jejuni being the most frequently identified trigger [3]. Molecular mimicry between microbial antigens and neural components leads to the production of anti-ganglioside antibodies, which mediate nerve injury through complement activation and macrophage infiltration [4].

While the majority of patients present with symmetrical ascending weakness and hyporeflexia, several clinical variants have been described. Miller Fisher syndrome (MFS), characterized by the triad of ophthalmoplegia, ataxia, and areflexia, accounts for approximately 5% of cases in Western populations. However, it is more prevalent in Southeast Asia countries like India, where it accounts for 19% to 25% of GBS cases [5]. Bickerstaff brainstem encephalitis (BBE) shares clinical features with MFS but additionally includes impaired consciousness [6]. Pharyngeal-cervical-brachial weakness represents another regional variant that may mimic brainstem pathology [7]. Cerebrospinal fluid (CSF) analysis and anti-GQ1b IgG antibody testing are important in diagnosing GBS and its variants, particularly MFS. CSF analysis helps identify albuminocytological dissociation (elevated protein with normal cell count), while anti-GQ1b IgG antibodies are highly sensitive in MFS, aiding in its diagnosis, and can also be present in other GBS variants [5-7]. These atypical presentations frequently lead to diagnostic delays, particularly when they occur in combination as overlap syndromes.

We present a diagnostically challenging case of GBS that initially manifested as isolated upper limb weakness before progressing to a severe overlap syndrome encompassing features of AIDP, MFS, and BBE. This case highlights the importance of considering atypical presentations of GBS, the value of serial electrophysiological studies, and the need for prompt immunotherapy to optimize outcomes.

## Case presentation

A previously healthy 60-year-old male presented to our emergency department with a 24-hour history of severe left shoulder pain and progressive weakness of the left upper limb. He reported no recent febrile illnesses, vaccinations, or trauma. On initial examination, the patient was afebrile with normal vital signs. Neurological assessment revealed Medical Research Council (MRC) grade 3/5 strength in the left deltoid and biceps muscles, with preserved strength in all other muscle groups. Deep tendon reflexes were diminished at the left biceps and brachioradialis but otherwise normal. Sensory examination was unremarkable, and cranial nerves were intact.

**Figure 1:**
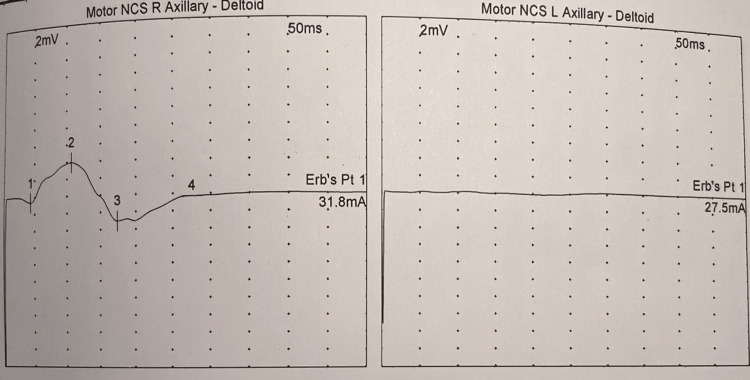
Initial motor NCS demonstrating axillary nerve involvement (A) Right axillary nerve study recording from the deltoid shows reduced CMAP amplitude (2 mV) with normal latency (50 ms) and elevated stimulation current at Erb's point (31.8 mA). (B) Left axillary nerve reveals similar findings (CMAP 2 mV, latency 50 ms, Erb's point stimulation 27.5 mA). These findings initially suggested bilateral axonal plexopathy before progression to generalized demyelination. CAMP, compound muscle action potential; NCS, nerve conduction studies

**Figure 2:**
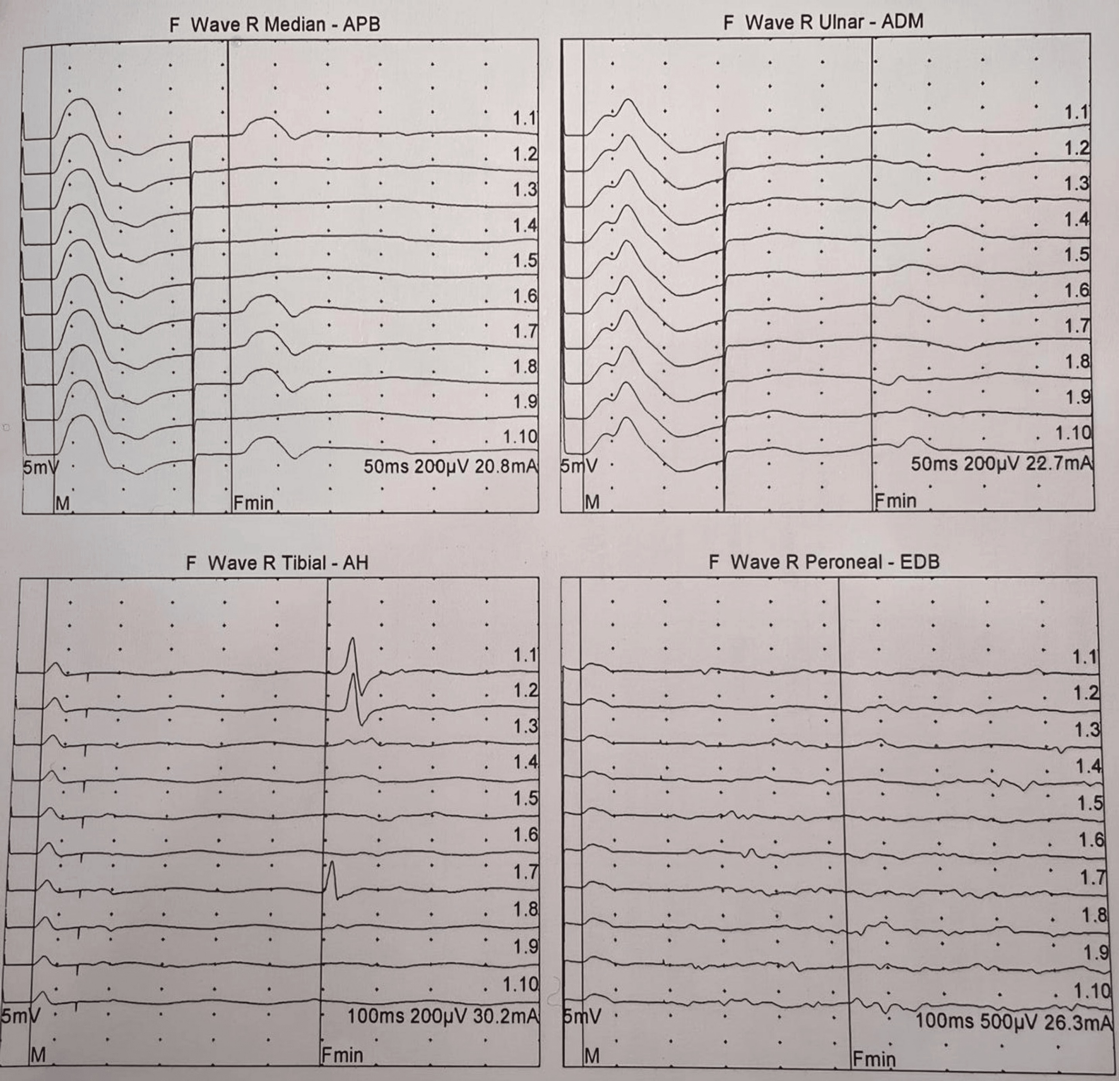
Serial F-wave studies demonstrating demyelinating features (A-D) F-wave studies performed on day 3 show absent/minimal F-waves (Fmin) in right median nerve (recording abductor pollicis brevis, stimulation 20.8 mA), right ulnar nerve (recording abductor digiti minimi, stimulation 22.7 ma), right tibial nerve (recording abductor hallucis, stimulation 30.2 mA), and right peroneal nerve (recording extensor digitorum brevis, stimulation 26.3 mA). The widespread absence of F-waves with normal CMAP amplitudes confirms acute inflammatory demyelination. CAMP, compound muscle action potential

Initial nerve conduction studies performed on day 1 demonstrated reduced compound muscle action potential (CMAP) amplitude (3.2 mV) in the left axillary nerve with normal sensory nerve action potentials, suggesting a possible axonal brachial plexopathy (Figures 1, 2). However, the clinical picture evolved dramatically over the subsequent 48 hours. By hospital day 2, the patient developed weakness in the right upper limb (MRC grade 4/5), and on day 3, he progressed to quadriparesis with involvement of both lower extremities (MRC grade 2-3/5). Concurrently, he developed dysphagia and dysarthria, necessitating repeat electrophysiological evaluation.

The follow-up nerve conduction studies on day 3 revealed characteristic features of acute inflammatory demyelinating polyneuropathy, including prolonged distal latencies (median nerve: 6.8 ms), conduction block (40% reduction in CMAP amplitude), and absent F-waves. CSF analysis showed albuminocytologic dissociation with elevated protein (120 mg/dL) and normal white cell count (2 cells/μL). The patient's condition continued to deteriorate, culminating in flaccid quadriplegia (MRC grade 0/5) and respiratory failure requiring mechanical ventilation by the evening of day 3.

On day 4, the patient developed complete external ophthalmoplegia with preserved vertical eye movements. Anti-GQ1b antibodies returned positive, supporting a diagnosis of MFS. By day 5, he exhibited progressive encephalopathy with a Glasgow Coma Scale score of 8/15, consistent with BBE. The composite clinical picture fulfilled the diagnostic criteria for an overlap syndrome combining AIDP, MFS, and BBE. All investigations done are summarized in Table 1.

**Table 1:** Comprehensive hematological, biochemical, and immunological laboratory profile Reference ranges indicate standard normal values for adults; age/sex-specific variations may apply. Bilirubin is measured as total, direct, and indirect (mg/dL), and Vitamin D3 is measured as 25-hydroxyvitamin D (nmol/L). Negative results for infectious markers (dengue, typhoid, malaria) rule out acute infection. Normal ABG and urine routine suggest no immediate metabolic or renal disturbances. ABG, arterial blood gas; ALP, alkaline phosphatase; ALT, alanine aminotransferase; AST, aspartate aminotransferase; CPK, creatine phosphokinase; CRP, C-reactive protein; ESR, erythrocyte sedimentation rate; GGT, gamma-glutamyl transpeptidase; Hb, hemoglobin; INR, international normalized ratio; LDH, lactate dehydrogenase; MCV, mean corpuscular volume; PCV, packed cell volume; PT, prothrombin time; WBC, white blood cells

Test	Result	Normal reference range
ESR	34 mm/hr	0–9 mm/hr
Hemoglobin	12.7 g/dL	13–18 g/dL
Total WBC count	9,300 /µL	4,000–10,000 /µL
PCV	38.1%	41–51%
MCV	87.79 fL	80–97 fL
Platelet count	138 × 10³/µL	150–450 × 10³/µL
Neutrophils	75%	40–70%
Lymphocytes	19%	20–40%
PT	21 seconds	Control: 14 seconds
INR	1.56	1.1
HbA1c	6.8%	4.2–6.2%
CRP	4.6 mg/L	Normal: <6.0 mg/L
Rheumatoid factor	11.3 IU/mL	Normal: <20 IU/mL
Total bilirubin	2.1 mg/dL	0–0.1 mg/dL
Direct bilirubin	0.97 mg/dL	0–0.3 mg/dL
Indirect bilirubin	1.13 mg/dL	1.1–1.35 mg/dL
ALP	130 IU/L	53–128 IU/L
Serum ammonia	112 µmol/L	12–98 µmol/L
Blood urea	37 mg/dL	6–44 mg/dL
Serum calcium (total)	9 mg/dL	8.4–10.4 mg/dL
Serum calcium (ionic)	1.04 mmol/L	1.1–1.35 mmol/L
Serum creatinine	1 mg%	0.9–1.2 mg%
CPK	254 IU/L	24–1950 IU/L
GGT	24 IU/L	0–45 IU/L
LDH	120 U/L	80–285 U/L
Serum total proteins	7.1 g/dL	6–8 g/dL
Serum albumin	3.4 g/dL	3.5–5.2 g/dL
Serum globulin	3.7 g/dL	2.3–3.5 g/dL
AST	68 IU/L	10–50 IU/L
ALT	74 IU/L	10–50 IU/L
Serum uric acid	7.1 mg/dL	3.4–7 mg/dL
Serum potassium	3.6 mEq/L	3.5–5.5 mEq/L
Vitamin D3 assay	69.4 nmol/L	75–250 nmol/L
Dengue NS1 antigen	Negative	–
Typhidot	Negative	–
Card test for malaria	Negative	–
ABG	Normal	–
Urine routine	Normal	–

Treatment with intravenous immunoglobulin ([IVIG] 0.4 g/kg/day for 5 days) was initiated on day 3. The patient underwent tracheostomy on day 20 due to prolonged ventilator dependence. Gradual neurological improvement began on day 15, with return of spontaneous eye movements followed by recovery of consciousness. He was successfully weaned from mechanical ventilation by day 25 and transferred to the general ward on day 27. At discharge on day 30, the patient had recovered to MRC grade 3/5 strength in all limbs with intact cognition. The patient’s clinical course, summarized in Table 2, illustrates the rapid progression from focal weakness to quadriplegia and brainstem involvement.

**Table 2:** Timeline of clinical progression and interventions CMAP, compound muscle action potential; CSF, cerebrospinal fluid; IVIG, intravenous immunoglobulin; MRC, Medical Research Council; NCS, nerve conduction studies

Hospital day	Clinical features	Diagnostic findings	Interventions
Day 1	Left arm weakness (MRC 3/5)	NCS: left axillary nerve CMAP ↓ (3.2 mV)	Analgesics, monitoring
Day 2	Right arm weakness (MRC 4/5)	-	Neurology consult
Day 3	Quadriparesis, dysphagia, respiratory failure	NCS: demyelination (prolonged latencies, absent F-waves); CSF: albuminocytologic dissociation	IVIG initiated, intubation
Day 4	Ophthalmoplegia	Anti-GQ1b Ab (+)	Continued IVIG
Day 5	Encephalopathy (GCS 8/15)	MRI brain: Normal	EEG (ruled out seizures)
Day 15	Return of eye movements	-	Tracheostomy
Day 25	Ventilator weaned	NCS: partial improvement	Aggressive rehab
Day 30	Discharged (MRC 3/5)	-	Outpatient follow-up

## Discussion

This case illustrates several clinically important aspects of GBS and its variant forms. The initial presentation with isolated unilateral upper limb weakness represents an exceptionally rare manifestation of GBS, occurring in less than 5% of cases [8]. Such atypical presentations frequently lead to misdiagnosis, particularly when early electrophysiological studies suggest focal pathology. Our patient's serial nerve conduction studies demonstrate the dynamic nature of GBS, with initial findings mimicking axonal plexopathy that subsequently evolved to show definitive demyelinating features. This underlines the importance of repeating electrophysiological studies when clinical suspicion for GBS remains high despite inconclusive initial results [9]. As highlighted in Table 3, the differential diagnosis for atypical GBS includes compressive neuropathies, brainstem ischemia, and neuromuscular junction disorders.

**Table 3:** Differential diagnosis of atypical GBS presentations CSF, cerebrospinal fluid; DWI, diffusion-weighted imaging; GBS, Guillain-Barré syndrome; NCS, nerve conduction studies

Clinical feature	GBS mimics	Distinguishing clues	Confirmatory tests
Asymmetric limb weakness	Cervical radiculopathy	No sensory level, progression to areflexia	NCS (demyelination), CSF analysis
	Brachial plexopathy	Rapid bilateral spread, bulbar involvement	Serial NCS
Ophthalmoplegia + encephalopathy	Brainstem stroke	Absent DWI changes on MRI	MRI, anti-GQ1b Ab
	Myasthenia gravis	Fatigability, response to edrophonium	Repetitive nerve stimulation
Quadriparesis + respiratory failure	Botulism	Pupillary dilation, descending paralysis	Stool toxin assay

The development of ophthalmoplegia and encephalopathy in our patient highlights the spectrum of anti-GQ1b antibody-associated disorders. These antibodies, which target gangliosides concentrated in the oculomotor nerves and brainstem, are present in more than 90% of MFS cases and approximately two-thirds of BBE cases [10]. The clinical distinction between these syndromes has become increasingly blurred, with many experts considering them part of a continuous spectrum rather than distinct entities [11]. Our case supports this conceptual framework, demonstrating a clear overlap between what have traditionally been classified as separate disorders.

Therapeutic decision-making in overlap syndromes presents unique challenges. While both IVIG and plasma exchange have demonstrated efficacy in classic GBS, data specific to overlap variants remain limited [12]. Therapeutic approaches for overlap syndromes, summarized in Table 4, prioritize IVIG but may incorporate emerging therapies in refractory cases. The presence of brainstem involvement in our patient raised theoretical concerns about autonomic instability during plasma exchange, leading to the selection of IVIG as first-line therapy. Emerging treatments such as eculizumab, a monoclonal antibody targeting complement protein C5, have shown promise in refractory cases and may be particularly relevant in anti-GQ1b-positive patients given the complement-mediated pathogenesis; however, these newer drugs will still take time to become the standard of care [13].

**Table 4:** Evidence-based management of GBS overlap syndromes GBS, Guillain-Barré syndrome; IVIG, intravenous immunoglobulin

Therapeutic option	Mechanism	Considerations for overlap syndromes
IVIG (2 g/kg over 5 days)	Neutralizes autoantibodies	Preferred in hemodynamic instability
Plasma exchange	Removes pathogenic antibodies	Avoid in autonomic dysfunction
Eculizumab (anti-C5)	Complement inhibition	Reserved for anti-GQ1b+ refractory cases
Corticosteroids	Immunosuppression	Not recommended
Supportive care (ventilation)	Prevents complications	Early tracheostomy if prolonged intubation

Prognostically, overlap syndromes typically follow a more protracted course than classic GBS. While most patients eventually achieve good functional recovery, the median time to independent ambulation may extend to six months compared with three months in typical cases [14]. Our patient's relatively rapid weaning from mechanical ventilation (25 days) compares favorably with published data on ventilated GBS patients, who average 35 days of respiratory support [15]. This may reflect the benefits of early immunotherapy initiation in our case.

## Conclusions

This case report describes an unusual presentation of GBS featuring an overlap between acute inflammatory demyelinating polyneuropathy, MFS, and BBE. The patient's initial manifestation as isolated unilateral upper limb weakness, followed by rapid progression to quadriplegia, respiratory failure, and brainstem dysfunction, underscores the diagnostic challenges posed by atypical GBS variants. Serial electrophysiological studies proved essential in establishing the correct diagnosis, while prompt immunotherapy likely contributed to the favorable outcome. Clinicians should maintain a high index of suspicion for GBS even in cases with unusual initial presentations, particularly when neurological deficits progress rapidly or involve multiple anatomical regions.
